# Hybrid Hydrogel Supplemented with Algal Polysaccharide for Potential Use in Biomedical Applications

**DOI:** 10.3390/gels11010017

**Published:** 2024-12-30

**Authors:** Dana Stan, Andreea-Cristina Mirica, Sorin Mocanu, Diana Stan, Iunia Podolean, Natalia Candu, Magdi El Fergani, Laura Mihaela Stefan, Ana-Maria Seciu-Grama, Ludmila Aricov, Oana Brincoveanu, Carmen Moldovan, Lorena-Andreea Bocancia-Mateescu, Simona M. Coman

**Affiliations:** 1 DDS Diagnostic, 031427 Bucharest, Romania; dana_stan@ddsdiagnostic.com (D.S.); research.imuno@ddsdiagnostic.com (A.-C.M.); proteomics@ddsdiagnostic.com (S.M.); dianastan335@gmail.com (D.S.); 2 ENT Department, “Maria Sklodowska Curie” Children’s Emergency Hospital, 077120 Bucharest, Romania; 3 Department of Inorganic Chemistry, Organic Chemistry, Biochemistry and Catalysis, Faculty of Chemistry, University of Bucharest, 4-12 Regina Elisabeta Blvd., 030016 Bucharest, Romania; iunia.podolean@chimie.unibuc.ro (I.P.); natalia.candu@chimie.unibuc.ro (N.C.); magdi.belkassim@unibuc.ro (M.E.F.); 4 Department of Cellular and Molecular Biology, National Institute of Research and Development for Biological Sciences, 296 Splaiul Independentei, 060031 Bucharest, Romania; laura.stefan@incdsb.ro (L.M.S.); anamaria.seciu@incdsb.ro (A.-M.S.-G.); 5 “Ilie Murgulescu” Institute of Physical Chemistry, Romanian Academy, 202 Spl. Independentei, 060021 Bucharest, Romania; laricov@icf.ro; 6 National Institute for Research and Development in Microtechnologies, 126 A Erou Iancu Nicolae, 077190 Voluntari City, Romania; oana.brincoveanu24@gmail.com (O.B.); carmen.moldovan@imt.ro (C.M.)

**Keywords:** hydrogel, wound, healing, dressing, Ulvan, algae

## Abstract

Hydrogels are a viable option for biomedical applications due to their biocompatibility, biodegradability, and ability to incorporate various healing agents while maintaining their biological efficacy. This study focused on the preparation and characterization of novel hybrid hydrogels enriched with the natural algae compound Ulvan for potential use in wound dressings. The characterization of the hydrogel membranes involved multiple methods to assess their structural, mechanical, and chemical properties, such as pH measurements, swelling, moisture content and uptake, gel fraction, hydrolytic degradation, protein adsorption and denaturation tests, rheological measurements, SEM, biocompatibility testing, and scratch wound assay. The hydrogel obtained with a higher concentration of Ulvan (1 mg/mL) exhibited superior mechanical properties, a swelling index of 264%, a water content of 55%, and a lower degradation percentage. In terms of rheological properties, the inclusion of ULV in the hydrogel composition enhanced gel strength, and the Alginate + PVA + 1.0ULV sample demonstrated the greatest resistance to deformation. All hydrogels exhibited good biocompatibility, with cell viability above 70% and no obvious morphological modifications. The addition of Ulvan potentiates the regenerative effect of hydrogel membranes. Subsequent studies will focus on encapsulating bioactive compounds, investigating their release behavior, and evaluating their active biological effects.

## 1. Introduction

Although there have been serious advances in wound dressings and smart patches, the wound care system still lacks solutions for chronic wounds, such as foot ulcers, venous leg ulcers, and pressure ulcers [1]. Some of the most recent reports have revealed that chronic wounds affect almost 2.5% of the total U.S. population, while in Europe, between 1.5 and 2 million people suffer from acute or chronic wounds [2,3]. Among the various options available for wound dressing (hydrocolloids, alginates, foams, and films), hydrogels seem to possess most of the desired characteristics: they can absorb large quantities of exudate, are biocompatible, are comfortable, and various healing agents can be incorporated into them while retaining their biological effect. Some efforts have been made to improve the mechanical properties of hydrogels by blending nanoclays with polydopamine and polyacrylamide [4].

Polyvinyl alcohol (PVA) is a biodegradable synthetic polymer used in the bio-technological field for the development of wound dressings, as carriers for certain therapeutic drugs, or in the field of tissue regeneration, with good results because of its biodegradability, biocompatibility, non-toxicity, and low cost [5]. PVA has been coupled with various compounds for the development of hybrid hydrogels, such as carboxymethyl cellulose, chitosan, honey, cellulose, nano silver, aloe polysaccharide, alginate, laponite, etc., which confer the material improved mechanical properties and the capacity to deliver various active compounds at the wound site, while maintaining the essential non-toxic, hydrophilic, and biocompatible properties [6,7,8,9,10,11].

Recently, algal polysaccharides (alginate, agarose, carrageenan, fucoidan, Ulvan, laminarin, porphyrin, starch, and cellulose) have received great attention due to their biocompatibility and biodegradability and their many beneficial effects, such as anti-wrinkle properties, UV protection, and antioxidant, anti-inflammatory, antimicrobial, and antiviral effects. Besides their many properties as active substances, polysaccharides originating from algae can determine high swelling capacity and crosslinking structure modifications of hydrogels, with effects on the mechanical and viscoelastic properties, which can be beneficial for applications involving drug delivery and wound healing [12].

Alginate is a natural polysaccharide that originates from brown algae (e.g., *Ascophyllum nodosum*, *Ecklonia maxima*, *Eisenia bicyclis*, *Macrocystis pyrifera*, and *Laminaria* spp.) and has been successfully used to develop wound dressings, mainly because of its efficient exudate absorption and ability to induce fibroblast multiplication, collagen synthesis, and the generation of granulation tissue [13].

Mixed hydrogels containing PVA and alginate have been developed for different applications, mostly for use as injectable material, drug delivery systems, and cell carriers, blending them with materials such as reduced graphene oxide (r-GO), hydroxy-apatite nanoparticles (HAP), polycaprolactone (PCL) microspheres, and chitosan [14,15,16,17,18].

Ulvan is a sulfated polysaccharide that mainly consists of rhamnose, iduronic acid, xylose, and glucuronic acid. It is found in the cell walls of *Ulva* spp. and has been shown to have various biological activities, such as antioxidant, anti-inflammatory, anticoagulant, and immunomodulatory properties, as well as antiviral effects [19]. There are a series of methods for the extraction and purification of Ulvan reported in the literature, and the general conclusion is that three main factors can affect yield, structure, and purity: temperature, pH, and extraction time [20]. One of the limitations of Ulvan hydrogels is poor mechanical strength, and a few studies have focused on improving these properties by combining Ulvan with various chemical compounds and polymers [21]. Various combinations, such as Ulvan–chitosan, Ulvan–methacrylate, Ulvan–NIPAAm, and Ulvan-tyramine-HRP have been developed for applications in tissue engineering, wound healing, or cell delivery. However, to our knowledge, no study has reported the use of alginate/PVA/Ulvan composite for use in biomedical applications. Our results showed that the developed hydrogel not only has improved mechanical properties, a desired feature in Ulvan hydrogels, but is also biocompatible and can induce proliferation in NCTC and HDF cells after 24 h exposure at all Ulvan concentrations.

The aim of our study was to develop a new and improved alginate and PVA hydrogel (Alg + PVA) by the incorporation of the algal polysaccharide and to evaluate this composite for structural, morphological, and biological effects for its potential use as a wound dressing material. To the best of our knowledge, this is the first attempt to in-corporate Ulvan into an Alg + PVA gel for this application. The obtained hydrogels, with various concentrations of Ulvan (0.1 mg/mL, 0.5 mg/mL, and 1 mg/mL), have been investigated by various methods (physical and morphological analysis; rheological testing; biocompatibility studies), and we found that the addition of Ulvan has improved the hydrogel mechanical properties, the water content, and swelling index and decreased the degradation percentage by 3–4%. In addition, the sample with the largest concentration of Ulvan exhibited increased resistance to deformation and excellent gel properties, as revealed by the analysis of the elastic and viscous modules.

## 2. Results and Discussion

### 2.1. Ulvan Extraction and Characterization

Compared to the majority of polysaccharides, Ulvan offers several advantages, such as water solubility, the ability to promote cell proliferation due to its high rhamnose content, and other notable properties, including antioxidant, antimicrobial, and immunomodulatory activity. However, the extraction and purification methods must be carefully tailored to the intended application, as certain properties, such as antioxidant activity or molecular weight integrity, may be compromised during processing. Because the algal habitat and the harvesting period can also influence composition and bioactivity, they must also be properly selected in order to ensure reproducibility of the material for biomedical applications, such as wound dressings (Table 1).

Hydrothermal extraction of the algal samples allowed the isolation of Ulvan with a yield of 21% *w*/*w*. The results of the elemental analysis (Table 2) are consistent with those reported in the literature [20]. Relatively high amounts of nitrogen (3.3%) and sulfur (4.2%) were observed.

Among the carbohydrates determined in the Ulvan hydrolysates, rhamnose and glucuronolactone were the most common, accounting for more than 73%. Glucose and other soluble sugars were detected in significantly lower amounts, with percentages varying from 1% to 11.3% (Table 3). These results are consistent with the literature data, which show that rhamnose is the predominant monomer, regardless of the extraction method used [44].

**Figure 1:**
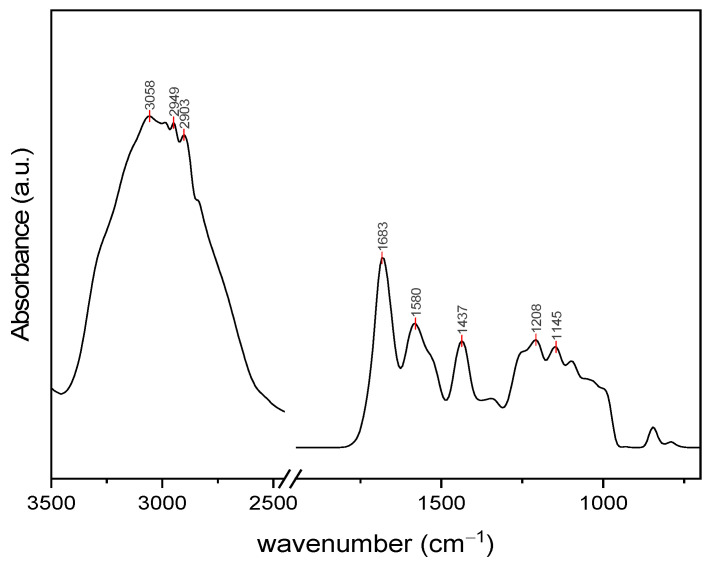
DRIFT spectrum of extracted Ulvan.

The DRIFT spectrum of the obtained Ulvan is presented in Figure 1, showing some of the characteristic bands (1537, 1253, 1100, and 846 cm^−1^) for this type of polysaccharide. The bands specific to carbohydrates are mainly present in two regions: 1160–990 cm^−1^, characteristic of carbonyl groups, and 950–800 cm^−1^, specific for sulfated carbohydrates. In the Ulvan spectrum, the band around 1630 cm^−1^ is attributed to the asymmetric stretching vibration of the C=O group, and that at 1437 cm^−1^ to the symmetric stretching of the same group from the carboxylic moieties of uronic acids [45]. However, the shift of the intense band in this region to 1683 cm^−1^ may indicate some conjugated C=O aldehyde groups as well as some residual proteins. This finding was confirmed by the additional band at 1580 cm^−1^. A sulfated moiety in the Ulvan structure is evidenced by several bands at 1255 cm^−1^ and 846 cm^−1^ belonging to S=O and C–O–S stretching vibrations [46,47]. The peaks in the region 1245–990 cm^−1^ correspond to C–O stretching vibrations, characteristic of glycosidic bonds between sugar monomers and uronic acid residues [46]. The common bands below 3000 cm^−1^ and broad shoulder at approximately 3200 cm^−1^ correspond to the stretching vibration of C–H in aliphatic residues and the stretching vibration of the O–H bond of the intermolecular and intramolecular hydrogen bonds, respectively [45,48].

**Figure 2:**
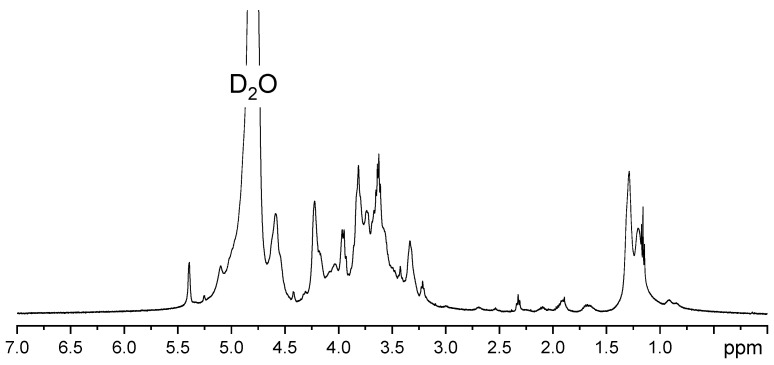
^1^H NMR spectrum of extracted Ulvan.

The proton NMR spectrum was specific to Ulvan, in agreement with the literature [49]. The 1H NMR spectrum shows specific signals in the 1–1.5 ppm region specific to the methyl groups of unsulfated and sulfated α-l-rhamnose (1.2 and 1.31 ppm). The 3.4–4.0 ppm region is representative of protons located on the monosaccharide ring and may indicate the presence of rhamnose, xylose, glucose, uronic acids, and galactose (Figure 2). For example, signals corresponding to xylose are indicated at 3.20 and 5.27 ppm [50]. The anomeric region (4.5–5.5 ppm) is assigned to uronic acids (β-d-glucuronic and α-l-iduronic acids) as well as to rhamnose (1,4-linked α-l-rhamnose) [51].

### 2.2. Physical and Mechanical Properties of the Hydrogel Membranes

#### 2.2.1. Variation of pH

There seems to be no indication that the addition of algal polysaccharides can induce pH variation. According to the results (Table 4), all samples had a pH value between 6 and 7.

#### 2.2.2. Swelling Index

**Figure 3:**
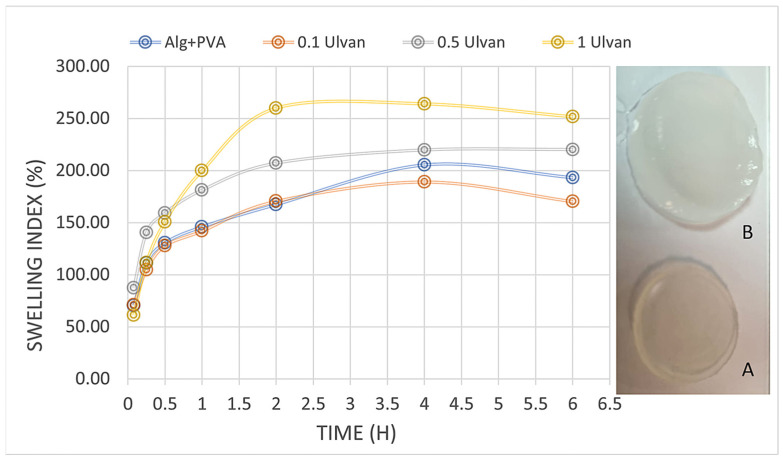
Swelling behavior of hydrogels over time: A—hydrogel before swelling in SWF; B—swollen hydrogel.

The samples without Ulvan swelled quickly but degraded significantly after only 4 h; at 6 h, they were impossible to weigh. All the Ulvan samples showed a constant stable swelling rate, with the highest percentage recorded for samples with 1 mg/mL, which reached 264% before degradation occurred (Figure 3). According to previous studies, two forces are involved in the swelling of hydrogels: one is the osmotic force, and the other is the elasticity force. Our results have revealed that the Alg/PVA hydrogel can absorb large quantities of simulated wound fluid in only about 4 h, when they reach a plateau phase and begin slow degradation. Biodegradability is another attractive feature of alginate gels, which means their use in the biomedical field will not further burden the ecosystem with hazardous waste [52]. Studies show that materials exhibiting water absorption of at least 100% in less than one day can influence the hemostasis stage and benefit the wound healing process [37].

Alginate-based wound dressings can absorb high amounts of wound exudate, maintaining an optimum humid environment for the wound, and do not adhere to the tissues, which is why their removal does not cause additional injury [53].

The swelling degree exhibits an increasing correlation with Ulvan concentration, aligning with the results reported by Sulastri et al., 2021 [32]. This parameter is intrinsically linked to hydrogels’ capacity to adsorb wound exudates, a critical property for effective wound management.

#### 2.2.3. Gel Fraction

**Figure 4:**
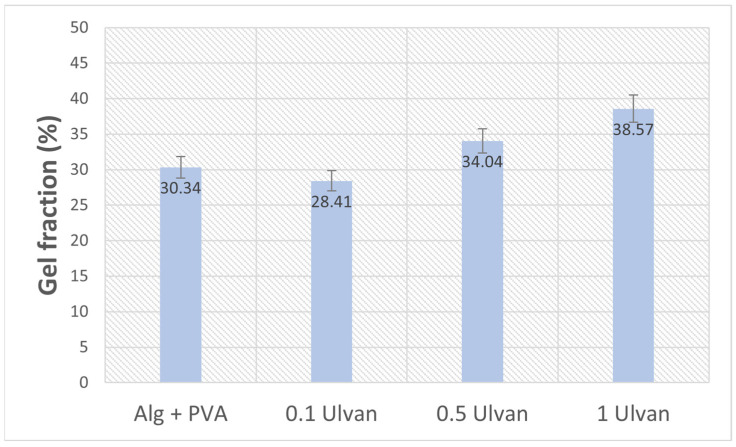
Gel fraction percentage of Ulvan-supplemented Alg + PVA hydrogels.

The results show that the integration of the algal polysaccharide determines a slight increase in the gel fraction, which is more obvious for the samples with 0.5–1 mg/mL Ulvan (Figure 4). The low gel fraction indicates that the components were mostly bound by physical interactions rather than covalent bonding. Physical crosslinking has certain advantages for biocompatibility because it renders them non-toxigenic and non-carcinogenic. Moreover, the hydrogels were prepared without using any toxic chemicals or toxic crosslinking agents or initiators [54]. Hydrogels obtained by physical bonding through phase separation and crystallization and ionic crosslinking (egg-box model of alginate and CaCl_2_) are highly pure and free of any other chemicals that can induce allergic reactions and complications, which is very desirable in applications that involve skin contact [55,56].

#### 2.2.4. Moisture Content and Uptake

**Figure 5:**
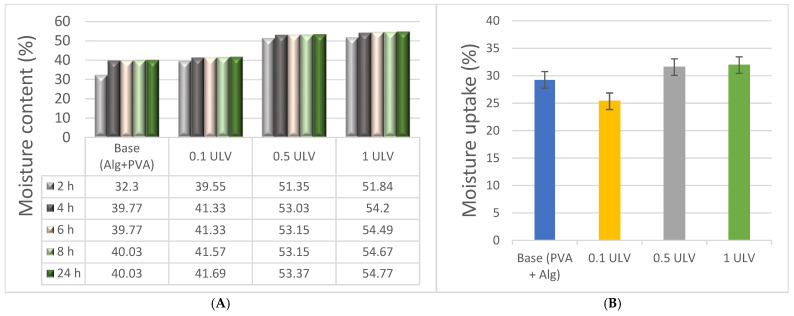
Moisture content (**A**) and uptake (**B**) of hydrogels.

As expected, an increase in the polysaccharide content enhanced the water retention of the hydrogels. Starting with the base (Alg + PVA), which has a water content of approximately 40%, the addition of Ulvan led to a continuous increase. Hydrogels containing 0.5 mg/mL and 1 mg/mL Ulvan exhibited water contents between 53% and 55% after 24 h of incubation (Figure 5). Although Ulvan contains a relatively hydrophobic compound, rhamnose, it also possesses numerous polar functional groups that may be responsible for retaining excess water molecules [20]. The water content of PVA/sodium alginate hydrogels aligns with the values reported in the literature, depending on the concentration of the compound [57]. Similarly, the water uptake was higher for the two hydrogel formulations with a higher Ulvan content, with a percentage exceeding 30%. The ability of hydrogels to maintain an appropriate water content is crucial for their intended application, which involves creating optimal conditions for wound healing, providing the necessary mechanical protection, and ensuring ease of application to wounds [58].

Compared to other Ulvan hydrogels, which achieved a maximum moisture content of 24% at a 5% Ulvan concentration, the moisture content of the hydrogels in this study increases with higher Ulvan concentrations, reaching 55% for the 1 ULV sample [32].

#### 2.2.5. Protein Adsorption

**Figure 6:**
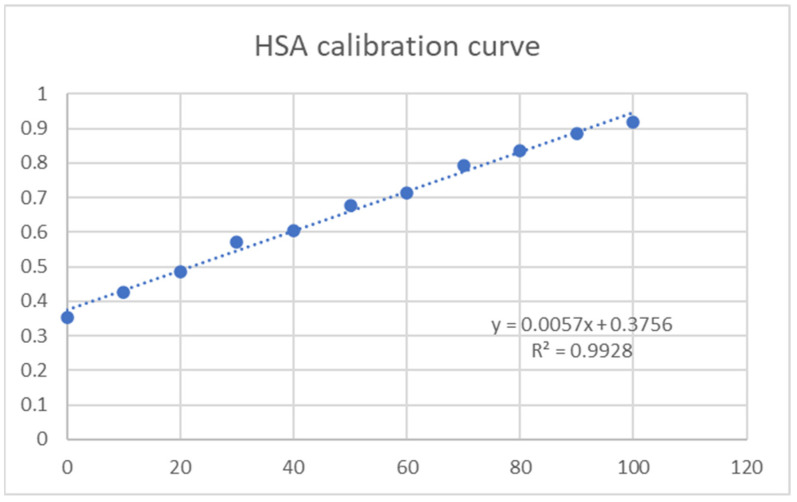
HSA calibration curve.

The hydrogel membranes were tested for protein adsorption efficiency using Human Serum Albumin (HSA) (73.05 µg/mL stock solution). To more accurately calculate the amount of HSA adsorbed, a calibration curve was obtained using concentrations between 0 and 100 µg/mL, and the results were obtained using a HITACHI UH5300 UV-VIS spectrophotometer (Figure 6).

Protein adsorption onto hydrogels is a critical process with implications in various fields, including biomaterials, drug delivery, and medical devices [59]. The adsorption mechanisms can be linked to electrostatic interactions, hydrogen bonding, hydrophobic forces, and specific binding sites on the hydrogel surface [60]. Protein adhesion significantly influences the biocompatibility of materials and can lead to the formation of a protein layer on the hydrogel surface, often termed a “biofoulinG″ layer, that can affect the interactions between the hydrogel and its biological environment, impacting biocompatibility, stability, and functionality.

**Figure 7:**
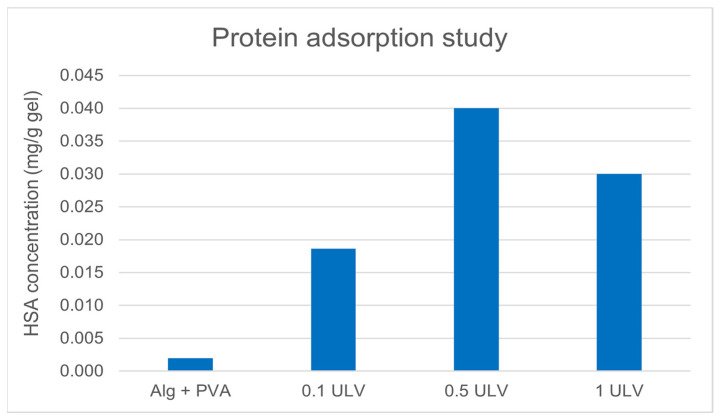
Protein adsorption comparison.

For wound healing dressings, to prevent microbial growth and reduce the risk of secondary infections, low protein adsorption of the hydrogel system is advantageous. Batch experiments were performed by adding a protein solution to the hydrogel samples. Using the calibration curve from HSA concentrations, we determined protein adsorption onto the hydrogel samples by reading the adsorption at 595 nm using UV-VIS spectrophotometry (Figure 7).

The results showed that the amount of protein adsorbed did not vary substantially among the three samples containing Ulvan.

#### 2.2.6. Inhibition of Protein Denaturation

**Figure 8:**
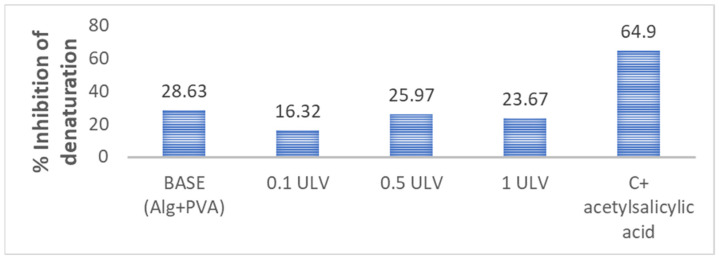
Hydrogels’ ability to protect against protein denaturation.

Our results revealed that the hydrogel membranes exhibited approximately the same capacity to protect proteins against thermal degradation, with percentages ranging from 16% to 28% as opposed to the aspirin control (64.9%), with variations unrelated to the addition of Ulvan (Figure 8). In another study, PVA and alginate hydrogels were tested for protein denaturation inhibition using an egg albumin model, and their results revealed similar percentages ranging from 18.95 to 23.98% [61]. Protection against protein denaturation, although not very pronounced, is a desired feature of wound dressing hydrogels because of their involvement in the structural and functional integrity of cells and their intracellular components. Protein denaturation is linked to the initiation and maintenance of inflammation [62].

#### 2.2.7. Hydrolytic Degradation

Hydrolysis occurs when polymer bonds interact with water molecules, resulting in cleavage of the chains into smaller fragments [63].

**Figure 9:**
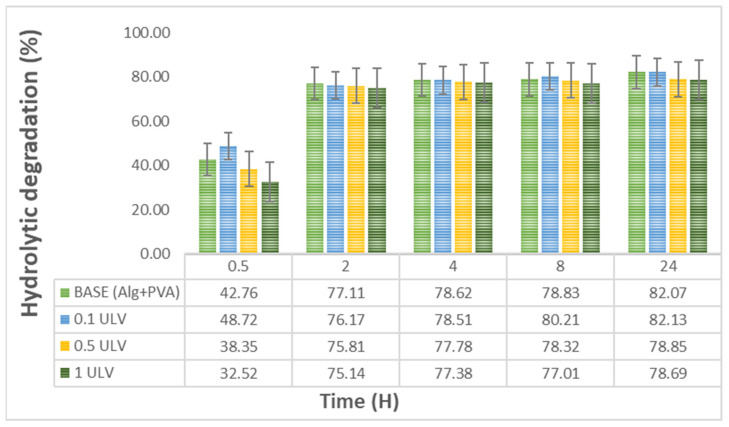
Hydrolytic degradation of the obtained hydrogels: Base (Alg + PVA), 0.1 mg/mL Ulvan (0.1 ULV), 0.5 mg/mL Ulvan (0.5 ULV), and 1 mg/mL Ulvan (1 ULV).

We found that in the first 30 min after water immersion, hydrogels undergo a rapid degradation phase, with 33–48% of the material degrading. Subsequently, between 2 and 24 h, the hydrogels displayed a significantly slower and more gradual increase in hydrolysis, reaching 80% (Figure 9). Similarly, in another study focused on developing hydrogel membranes for wound dressing applications, the PVA-sodium alginate (SA) hydrogel exhibited a weight loss of approximately 60% for SA concentration of 75% after approximately 24 h [64].

The addition of Ulvan had a minor effect on the degradation profile, which was more obvious between 8 h and 24 h. According to the specialized literature, susceptibility to degradation is important for cell proliferation and metabolism, as demonstrated by Thai and collaborators in a study regarding the influence of hydrogel degradation on the angiogenic and regenerative potential of cell spheroids [65]. Other studies have stated that degradable scaffolds can determine secretion of some very important factors in wound healing, such as matrix metalloproteinases (MMPs) and cytokines, while also triggering deposition of collagen, cell migration, and angiogenesis [66].

It is noteworthy that hydrogels with a higher concentration of Ulvan, specifically 0.5 mg/mL and 1 mg/mL, exhibited a lower degradation percentage than the base hydrogel or the hydrogel containing 0.1 mg/mL Ulvan. This may be attributed to the higher polysaccharide content, which contributed to the enhancement of the macromolecular network and higher water retention [67]. The hydrolysis rate can be adjusted by modifying parameters such as the crosslinking density, which is determined by the polymer concentration or degree of crosslinking during material fabrication [68].

### 2.3. Mechanical and Morphological Properties of the Hydrogel Membranes

#### 2.3.1. Rheological Studies

**Figure 10:**
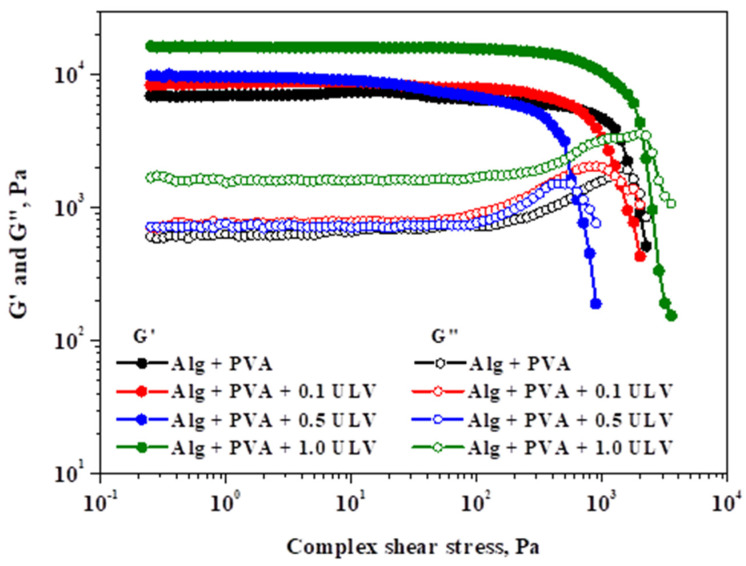
Analysis of elastic and viscous modules in relation to shear stress at 1 Hz oscillation frequency.

Figure 10 shows the impact of shear stress on the viscoelastic modules at a frequency of 1 Hz and temperature of 25 °C.

For the examined probes, the elastic modulus (G′) and viscous modulus (G″) showed linear viscoelastic behavior, followed by a decreasing pattern for both parameters. A decrease in rheological modules with shear stress marks the end of the LVER, indicating irreversible deformation of the gel’s structure and the appearance of the yield point. The yield stress (G″ ≥ G′) describes the elastic limit and the beginning of the gel’s plastic deformation. Therefore, the obtained critical points, together with G′ and G″ at 5.0 Pa, are listed in Table 5.

The tested hydrogels differ in terms of their strength (values of G′ and G″) and yielding characteristics, which were significantly influenced by the ULV content. The viscoelastic modules exhibit a positive correlation with the ULV content. Although the addition of ULV initially leads to a decrease in the yield stress, at the highest ULV concentration, it undergoes a significant increase compared to the other systems. Consequently, Alg + PVA + 1.0 ULV, which contained the highest quantity of ULV, exhibited the highest resistance to deformation. This is due to the fact that it possessed the longest LVER, the highest G′ and G″ values, and the greatest yield stress.

**Figure 11:**
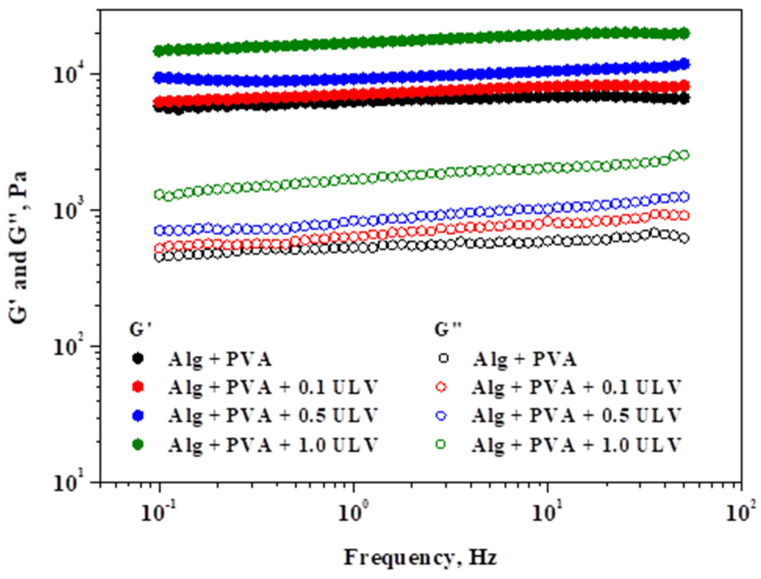
Analysis of elastic and viscous modules in relation to oscillation frequency under a shear stress of 5 Pa.

Figure 11 depicts the effect of the applied frequency on the viscoelastic modules of the investigated hydrogels while maintaining a constant shear stress.

The systems exhibited gel-like behavior, as indicated by the viscoelastic spectra (G′ > G″), and were consistent with those of the amplitude sweep stress. Over the entire frequency range that was applied, a gradual increase in the viscous modulus was observed, with the highest increase occurring at higher frequencies. Compared to the other modules, G′ was significantly less affected by the oscillation frequency. Examining the significant values of the two rheological modules and the minimal variations in the elastic modulus with respect to the applied frequency are characteristics that can be used to determine the gel strength. As a result, the Alg + PVA + 1.0 ULV material exhibits excellent resistance as a gel. The Alg + PVA + 0.5 ULV system followed this trend, while the Alg + PVA and Alg + PVA + 0.1 ULV systems showed comparable behavior and still exhibited good resistance to deformation. PVA addition confers properties such as tensile strength, surface hydrophilicity, and flexibility, all desired properties when selecting a material for wound dressing application [69]. Our results revealed that the PVA/Alg blend exhibits good resistance to deformation, which makes it a good candidate for wounds that are in hard-to-reach regions (armpits, leg/arm junctions, interdigital, etc.). In summary, the incorporation of ULV in the composition of hydrogel systems, even at low concentrations, resulted in an enhancement in the gel strength, which quantifies the stiffness of the polymer network.

#### 2.3.2. SEM Analysis

Macroscopic analysis of the hydrogels revealed small changes in their morphology, including a slight increase in thickness and a minor modification in color. Although visible to the naked eye, these changes did not affect the overall appearance of the gels. However, scanning electron microscopy (SEM) provided a more in-depth examination of the structural changes within the hydrogel network, revealing significant differences at the microscopic level, particularly with the incorporation of Ulvan.

At lower Ulvan concentrations, the hydrogel structure appeared relatively similar to that of the base material, with less pronounced network formation. However, as the Ulvan concentration increased to 0.5 mg/mL and 1 mg/mL, a more robust and tightly crosslinked network structure was formed. The SEM images clearly showed that at these higher concentrations, the hydrogels exhibited a denser and more interconnected polymer matrix, which could be attributed to the increased crosslinking between the hydrogel chains facilitated by Ulvan.

**Figure 12:**
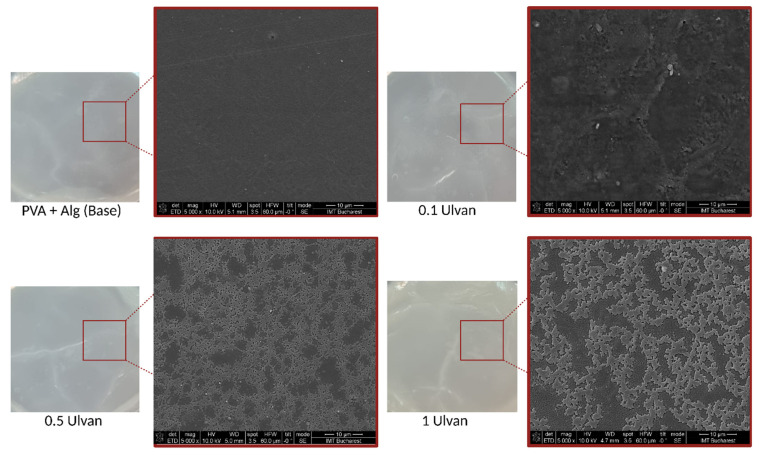
SEM micrographs for the base hydrogel and the Ulvan-supplemented variants.

This enhanced crosslinking not only modified the gel microstructure but also contributed to a noticeable increase in the mechanical strength of the hydrogel. A tighter polymer network distributes stress more effectively, making the material more resilient. These improvements are reflected in the increased firmness of the gels at higher Ulvan levels. Figure 12 highlights these changes, showing a clear difference between the base hydrogel and those with higher Ulvan concentrations, demonstrating the role of Ulvan in strengthening the matrix.

**Figure 13:**
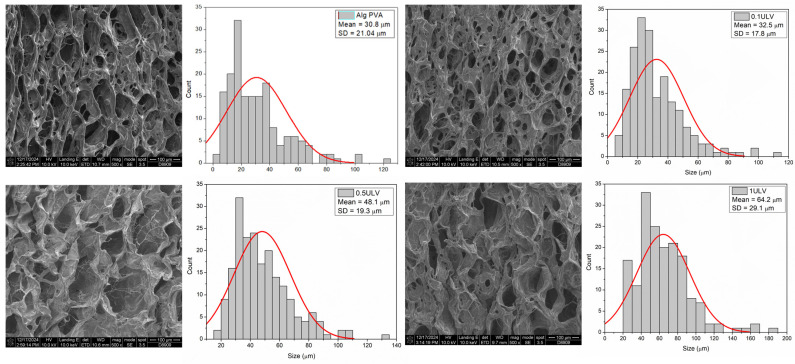
SEM images revealing the porous nature of the hydrogels and pore size quantitative evaluation.

The analysis of the lyophilized hydrogen membranes has provided critical insights into the microstructural changes induced by varying Ulvan content within hydrogels. It is evident that an increase in the Ulvan concentration significantly affects the internal architecture of the swollen hydrogel. Notably, an incremental addition of Ulvan results in a pronounced enlargement of pore sizes within the hydrogel matrix. The micropore distribution for samples including Alg-PVA, 0.1 ULV, 0.5 ULV, and 1 ULV was quantitatively assessed through SEM images by measuring approximately 200 micropores per sample. The software ImageJ 1.x (open-source) was employed to extract and analyze the data, enabling the construction of corresponding histograms. The measured micropore sizes ranged as follows: 3–120 µm (Alg-PVA), 7–112 µm (0.1 ULV), 16–131 µm (0.5 ULV), and 20–186 µm (1 ULV). Gaussian fitting of the histograms provided a robust characterization of the micropore distributions (Figure 13).

The Full Width at Half Maximum (FWHM) percentage for the micropores indicated that the majority fell within size ranges of 12–51 µm, 15–50 µm, 29–66 µm, and 35–90 µm for Alg-PVA, 0.1 ULV, 0.5 ULV, and 1 ULV samples, respectively, with mean diameters of 30.8 ± 21.0 µm, 32.5 ± 17.8 µm, 48.1 ± 19.3 µm, and 64.2 ± 29.1 µm (Table 6). According to the literature, the optimum pore size for mammalian skin regeneration is between 20 and 125 µm, which makes the variants 0.5 ULV and 1 ULV more suitable for wound dressings, since the majority of pores are ≥20 µm [70].

This observed phenomenon can be attributed to the intrinsic physico-chemical properties of Ulvan. Its hydrophilic nature and complex molecular architecture enhance water retention and interaction within the hydrogel network, promoting the formation of larger pores during the swelling process [12]. The results highlight the significance of Ulvan concentration as a key parameter in tuning the microstructural and functional properties of hydrogel-based materials, underscoring its potential for applications requiring tailored pore architectures.

The porous structure of hydrogels plays an important role in wound healing by facilitating the efficient transport of oxygen and nutrients to the wound site, which helps maintain an optimal microenvironment for tissue repair [42]. An interconnected pore network supports cell migration, enhances waste removal, and promotes the exchange of essential biomolecules, thereby accelerating the healing process [71]. Furthermore, the high porosity and controlled pore size distribution of the hydrogel membranes not only improve the filtration of contaminants and pharmaceutical agents but also support the encapsulation and controlled release of targeted drugs/enzymes or other therapeutic molecules [72,73]. This is particularly important, as diffusion is the primary mechanism for drug release from hydrogels.

### 2.4. Antimicrobial Analysis

**Figure 14:**
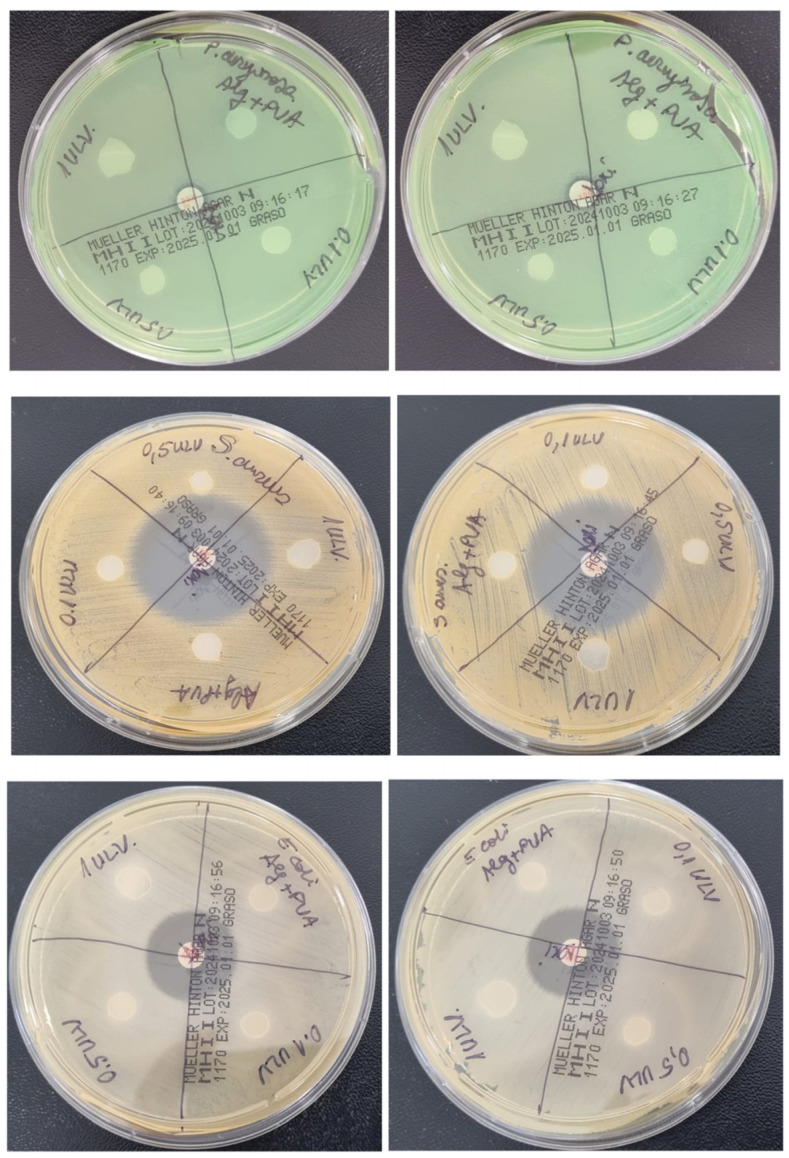
Antibacterial effect of Ulvan-supplemented hydrogel membranes against *P. aeruginosa*, *S. aureus*, and *E. coli*.

Because recent studies suggest a good antibacterial effect of Ulvan, we have selected both Gram-positive and Gram-negative strains for this evaluation (Figure 14). Our results show no antibacterial effect at the tested concentrations of Ulvan against *S. aureus* or *E. coli* strains, which is in line with another recent study [74]. In our study, there was also no inhibition zone for the *P. aeruginosa* strain, as opposed to other studies that reported an inhibition zone of about 12 mm but at higher concentrations, ≥25 mg/ mL [74,75].

### 2.5. Cytotoxicity Evaluation

**Figure 15:**
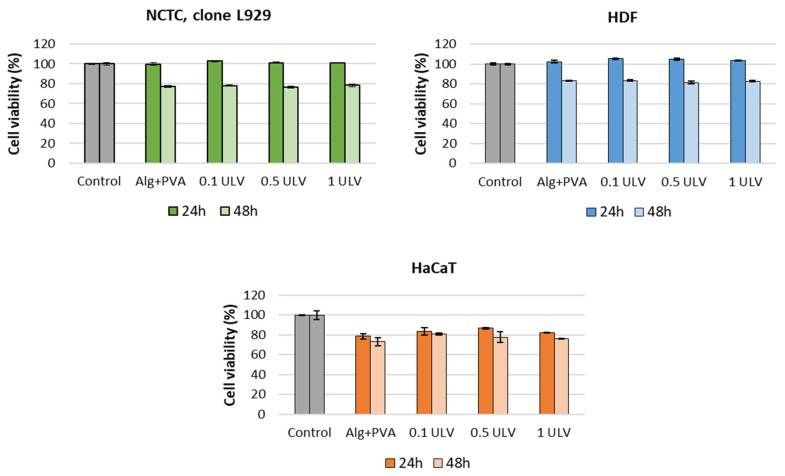
Viability of NCTC, clone L929, murine fibroblasts, HDF human dermal fibroblasts, and HaCaT human keratinocytes cultivated in the presence of the Ulvan-supplemented hydrogels for 24 h and 48 h, evaluated by the MTT assay. Samples were reported against the control (untreated cells), considered to have a 100% viability. Data were expressed as the average of three replicates (mean ± SD).

The potential cytotoxic effect of Ulvan-supplemented hydrogels was assessed in three cell lines (NCTC, clone 929, HDF, and HaCaT) using the MTT assay, which evaluates the activity of mitochondrial dehydrogenases. The MTT results for NCTC murine fibroblasts showed that all tested hydrogels exhibited no cytotoxic activity at either exposure time (24 and 48 h), and the percentages of cell viability were higher than 70% (Figure 15). After 24 h, the values of cell viability ranged between 99.8% for Alg + PVA and 102.89% for the 0.1 ULV hydrogel; after 48 h, the cell viability decreased, and the values were similar among samples (77.09% for Alg + PVA, 78.19% for 0.1 ULV, 76.44% for 0.5 ULV, and 78.45% for 1 ULV). Similar results were obtained when tested on HDF cells. Thus, the cell viability of hydrogels was higher compared to the control sample (100%) after 24 h, with values ranging between 102.53% for Alg + PVA and 105.3% for 0.1 ULV. After 48 h of treatment, the cell viability decreased around 80%, the values ranging between 81.52% for 0.5 ULV and 83.51% for 0.1 ULV. When hydrogels were tested on HaCaT keratinocytes, the obtained results showed cell viability values lower than those obtained for NCTC and HDF cells after 24 h of treatment but still within the cytocompatibility range (>70%). Thus, the percentages of cell viability ranged between 78.62% for Alg + PVA and 86.73% for 0.5 ULV. After 48 h, the values slightly decreased, ranging between 73.16% for Alg + PVA and 80.96% for 0.1 ULV (Figure 13). Although all hydrogels were cytocompatible, in general, cell viability increased when Ulvan was added to the Alg + PVA hydrogels.

**Figure 16:**
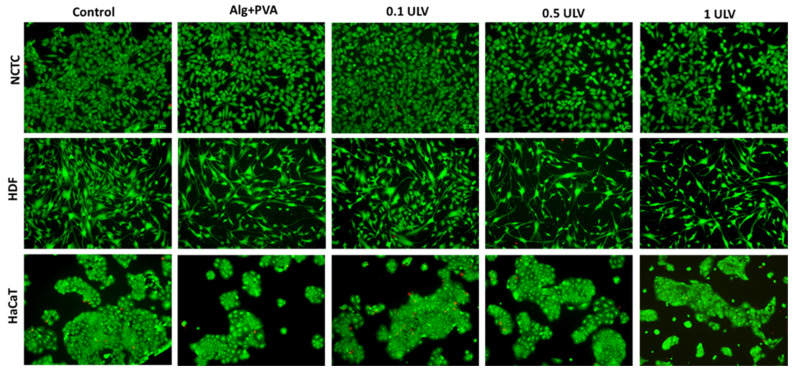
Fluorescent staining with calcein-AM (green) and ethidium homodimer-1 (red) of NCTC, HDF, and HaCaT live and dead cells, untreated (control) and treated with Alg + PVA, 0.1 ULV, 0.5 ULV, and 1 ULV hydrogels for 48 h. Scale bar = 50 μm.

The viability and morphology of the fibroblasts and keratinocytes cultivated in the presence of the hydrogels were also evaluated by fluorescence microscopy after staining the live cells with calcein (green) and dead cells with ethidium homodimer (red). After 48 h of treatment, all cell types maintained their viability, whereas the number of dead cells was negligible, indicating cytocompatibility of all tested hydrogels. No significant changes in cell morphology were observed and all cell types maintained their normal morphological appearance, similar to that of the control sample (Figure 16). However, the cell density was slightly reduced compared to the control, these results being in accordance with those obtained by the MTT quantitative assay.

### 2.6. Scratch Wound Evaluation

**Figure 17:**
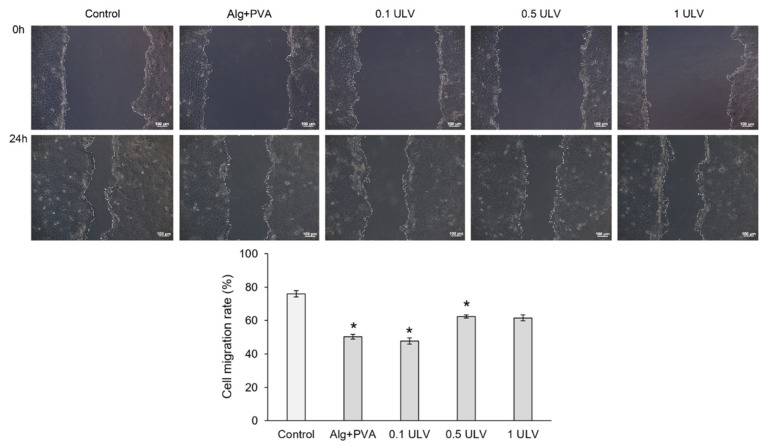
Microscopy images of the HaCaT monolayer after in vitro creation of a skin injury and treatment with the extraction medium of the Ulvan-based hydrogels, for 24 h; scale bar = 100 µm. Cell migration in the injured area can be observed. Migration rate (%) of HaCaT keratinocytes treated with Ulvan-based hydrogels, for 24 h, evaluated with ImageJ software. Results were expressed as mean value ± SD (n = 3). * *p* < 0.05 compared to the control (untreated cells).

An in vitro model of skin injury (scratch assay) was used to evaluate the ability of Ulvan-based hydrogels to accelerate the proliferation and migration of cells and to cover the injured area, and therefore to induce the healing of a “wound”. Our results showed that all tested samples induced cell proliferation and migration into the wounded area after 24 h of treatment, although cell migration rates were lower than that of the control (untreated cells) (76.03%) (Figure 17). Thus, 0.5 ULV and 1 ULV presented the highest cell migration rate (62.43% and 61.57%, respectively) and ability to induce wound healing, followed by Alg + PVA (50.29%) and 0.5 ULV (47.78%).

In conclusion, the 0.5 ULV and 1 ULV samples were the most effective in repairing the injured cell monolayer after 24 h of treatment, promoting keratinocyte proliferation and migration.

## 3. Conclusions

The newly developed hydrogel membranes were obtained through a dual crosslinking process by combining ionic bonding and freeze–thaw cycling, which provided the development of numerous bonding sites and a more compact network structure.

The incorporation of Ulvan significantly enhanced the performance of the hydrogel membranes. A higher crosslinking density generally leads to reduced swelling as the network becomes more rigid and less capable of absorbing water. However, hydrogels with 1 mg/mL Ulvan exhibited a higher swelling index (264%), which could be explained by the less homogenous distribution of the polysaccharide at the highest concentration, as revealed by the SEM micrographs. The hybrid hydrogels exhibited relatively low gel fractions (28–38%), likely due to the predominance of physical bonding interactions between the components.

The moisture content of the samples increased with an increase in the concentration of Ulvan, ranging from 40% to 55%. This variation is highly relevant when determining the most appropriate option for biomedical applications, enabling the selection of an optimal material tailored to the specific characteristics of the wound in personalized medicine.

A notable increase in protein adsorption was observed in hydrogels containing Ulvan compared to the base formulation of alginate and PVA, with the 0.5 ULV sample adsorbing the highest concentration of HSA. The inhibition of protein denaturation results showed that the hydrogels were able to protect proteins by between 16% and 28% against thermal degradation.

The incorporation of ULV into the hydrogel membranes, even at low concentrations, led to a notable improvement in gel strength, with samples containing 1 mg/mL ULV demonstrating superior elastic and viscous properties compared with those without ULV. These findings may be attributed to the high polysaccharide content, which contributes to the strengthening of the macromolecular network.

All tested hydrogels (with and without Ulvan) had good biocompatibility, as shown by the MTT assay, with viability rates above 70%. Fluorescence microscopy revealed that the addition of Ulvan did not induce any modifications in cell morphology, and viability was maintained among the cells from all the tested lines, regardless of the polysaccharide concentration.

Considering that the hydrogels demonstrated excellent properties in terms of swelling indices, water content, and viscoelasticity and exhibited good biocompatibility, they are suitable candidates for potential use in wound healing applications. Future research will focus on the encapsulation of bioactive compounds within the hydrogel matrix, with an in-depth investigation of their release kinetics under various physiological conditions. Additionally, the studies will evaluate the biological activity of the released compounds, including their potential effects on cell proliferation, tissue regeneration, and antimicrobial properties, to further assess their suitability for advanced biomedical applications.

## 4. Materials and Methods

### 4.1. Materials

Polyvinyl alcohol (PVA) (Mw-89–98,000), Potassium chloride (KCl), Phosphate buffer saline, bovine serum albumin (fraction V, 96%), acetylsalicylic acid, calcium chloride, ethanol (pure reagent >99.5%); HCl (pure reagent 37%); Na_2_CO_3_ (anhydrous, ≥99.5%, powder); H_2_SO_4_ (pure reagent 95.0–98.0%); NaOH (reagent grade, 97%, powder); KBr (anhydrous, free-flowing, ≥99%); and D_2_O (isotopic purity 99.8 atom % D) were supplied by Sigma Aldrich, St. Louis, MO, USA. Sodium alginate and sodium chloride were purchased from VWR Chemicals, Radnor, PA, USA. Anhydrous glycerol (99.0–101%) was purchased from Honeywell, Charlotte, NC, USA; distilled water from Rompack, Bucharest, Romania; human serum albumin from Alfa Aesar, Haverhill, MA, USA; and Pierce™ Coomassie (Bradford) Protein Assay Kits (Thermo Scientific, Waltham, MA, USA). Algal specimens of *Ulva rigida* were collected from the marine environment in region 2 Mai—Vama Veche, Constanta, Romania, in October 2022.

Cell lines murine fibroblast cells (NCTC, clone L929) were provided by ECACC, UK Health Security Agency, Porton Down, Salisbury, UK; human dermal fibroblasts (HDF) by Cell Applications inc, San Diego, CA, USA; and human keratinocytes (HaCaT) by AddexBio, San Diego, CA, USA.

*Staphylococcus aureus* (ATCC 700699), *Pseudomonas aeruginosa* (ATCC 10145), and *Escherichia coli* (ATCC 25922) strains were purchased from MediMark, Grenoble, France. Culture media were procured from Avena, Bucharest, Romania, and standardized doxycycline discs (30 μg) from Bio-Rad, Hercules, California, USA.

### 4.2. Ulvan Extraction and Characterization

Ulvan was extracted from dry algal powder in a Teflon-lined stainless steel hydrothermal autoclave. Solid algae and water were introduced into the reactor at a ratio of 6 g/100 mL and ultrasonicated for 10 min at 70 °C prior to the actual extraction. The autoclave was further heated at 121 °C for 30 min. The hot aqueous solution was filtered through cotton cloth, and the filtrate was allowed to cool at room temperature. The extraction was repeated, and the resulting filtrates were combined. The extracted polysaccharides were precipitated by the addition of 96% (*v*/*v*) ethanol (filtrate/EtOH ratio 1:1.5 *v*/*v*) [76]. The mixture was kept overnight in a refrigerator (4 °C). The resulting solid was centrifuged, washed three times with ethanol, and sonicated in an ultrasonic bath for 1 h in an acetone/MeOH (1:1 *v*/*v*) mixture to remove the remaining pigments. The obtained Ulvan was finally dried overnight under vacuum to afford Ulvan as an off-white powder.

#### 4.2.1. NMR Analysis

^1^H NMR analysis (Nuclear Magnetic Resonance Spectroscopy) of the Ulvan samples was performed with the Instrument Bruker AvanceIII equipment, (Carteret, NJ, USA) at a frequency of 500 MHz using D_2_O as a deuterated solvent.

#### 4.2.2. FTIR Analysis

FTIR spectra (Fourier Transform Infrared Spectroscopy) were recorded on a BrukerTenso-II FTIR spectrophotometer (Carteret, NJ, USA) adapted with a Smart Accessory for diffuse reflectance measurements (DRIFT) in the range of 500–4000 cm^−1^. Recorded spectra represent an average of 64 scans at 16 cm^−1^ resolution. Heterogeneous samples were mixed with KBr in a ratio of roughly 95:5 KBr:sample.

#### 4.2.3. Elemental Analysis

The elemental analysis was carried out on EuroVector Euro EA Elemental Analyzer (Shimadzu, Pavia, Italy). The combustion elemental analyzer allows to determine C, N, H, and S content in algae and Ulvan samples. Sulfanilamide has been used as a calibration standard.

#### 4.2.4. GC-MS Analysis

Identification of the monomers in hydrolyzed Ulvan was performed by GC-MS (gas chromatography–mass spectrometry). The GC-MS analysis was carried out with a THERMO Electron Corporation instrument (Thermo Scientific, Waltham, MA, USA) equipped with TG-5SilMS column 30 m × 0.25 mm × 0.25 μm. The injector port was set up at 250 °C. The temperature in the oven was maintained at 50 °C for 1 min and then increased to 250 °C at a rate of 7 °C min^−1^.

The distribution (Selectivity (%)) of the Ulvan components in the hydrolysates was calculated using the following equation, were *i* stands for product of interest and *p* for all products:

(1) $$S_{i}=\frac{{Yield}_{i}}{{Yield}_{p}}\times 100$$

#### 4.2.5. Acid Methanolysis of Ulvan

In order to obtain hydrolysates for chromatographic, analysis Ulvan was subjected to acid methanolysis. The method was based on the procedure of Sundberg et al. [77]. A quantity of 10 mg of Ulvan was weighed, and 2 mL of 2 M HCl solution in anhydrous methanol was added. The samples were stirred for 4 h at 100 °C, then cooled to room temperature. From each sample, 500 µL of solution was taken and 50 µL of pyridine was added to neutralize the acid; the samples were evaporated to dryness under vacuum. The reaction products were derivatized with a silanizing agent (50 µL pyridine, 150 µL BSTFA (N, O-bis(trimethylsilyl)trifluoroacetamide) and TMCS (trimethylchlorosilane) for 2 h at 70 °C, then the samples were diluted with 0.5 mL of ethyl acetate and analyzed chromatographically.

### 4.3. Hydrogel Membrane Synthesis

The PVA and sodium alginate base was obtained as previously described by Stan et al., with small modifications [78]. PVA was dissolved in distillated water and a 5% solution was obtained, which was kept at 100 °C until use. For the Ulvan-containing samples (0.1 mg/mL, 0.5 mg/mL, and 1 mg/mL), the algal polysaccharide was dissolved in water before adding the PVA. Then, a solution of 1.6% sodium alginate (*w*/*v* %) 0.1% CaCl_2_ (*w*/*v* %) and 12% (*w*/*v* %) glycerol was obtained in 50 mL distilled water. Briefly, alginate and CaCl_2_ were mixed in a clean, dried glass, glycerol was added, and they were mixed on a hot plate at 100 °C for 5 min at 600–700 RPM. After homogenization, 50 mL of distilled water was added, and the mixture was left to homogenize for 5–10 min before adding the PVA solution previously prepared. The mixture was poured in sterile Petri dishes, with a diameter of ~50 mm, and incubated at 30 °C for 24 h. The membranes were then transferred in a freezer at −30 °C for 24 h. Following these steps, the freezing–thawing (F–T) method reported by Stauffer and Peppast [79] was employed, which involved three cycles of two hours each (1 h freeze, 1 h thawing). After the final thawing, the hydrogel membranes were incubated in an oven at 50 °C for 24 h for final drying. In order to avoid microbiological contamination of the membranes, they were subjected to a UV sterilization cycle of 15–20 min and stored in sealed aluminum bags with desiccant until further use.

### 4.4. Hydrogel Membrane Physico-Chemical Characterization

#### 4.4.1. pH Variation Analysis

For the pH variation analysis, 1 g of each hydrogel membrane type was added in 10 mL PBS (pH~7.4), transferred to a hot plate, and kept until reaching 37 °C (temperature controlled using a Orion Star digital pH-meter, supplied by Thermo Fisher, Waltham, Massachusetts, United States). Then, samples were vortexed for 1 min and filtered so that large hydrogel pieces are discarded. The obtained solution was analyzed using a digital pH meter (Thermo Fisher Orion Star A211, Vantaa, Finland). Analysis was performed in triplicate, and the results are presented as mean ± standard deviation (S.D.).

#### 4.4.2. Swelling Index

The swelling index evaluation was conducted using the gravimetric method, in which artificial wound fluid was prepared according to the method described by Arafa [80]. Samples were cut using a sterile falcon tube with a diameter of ~1.5 cm, weighed (triplicate), immersed in 10 mL of simulated wound fluid (SWF), and left at room temperature (approx. 25 °C). Gels were weighed at different time intervals (5 min.; 15 min.; 30 min.; 1 h; 2 h; 4 h; 6 h; 24 h) after eliminating the excess fluid using a filter paper. Swelling index (*SI*%) was calculated using the following formula:

(2) $$SI\;\left(\%\right)=\frac{\left(Wf-Wi\right)}{Wi}\times 100$$

where *Wf* is the final weight at each tested time and *Wi* is the initial weight.

#### 4.4.3. Gel Fraction

Samples cut at approx. 1.5 cm diameter (three for each type of membrane) were weighed and then dried in an oven at 60 °C until they reached a constant weight (around 60 min), with weighing every 30 min. Then, they were immersed in 20 mL of PBS and incubated at 25 °C for 24 h. After incubation, samples were transferred into dry glass plates and dried in an oven at 60 °C until no moisture was observed (~2 h 30 min.). The samples were weighed again, and the gel fraction for each sample was calculated using the formula [81]:

(3) $$GF\;\left(\%\right)=\frac{Wg}{W0}\times 100$$

where *Wg* is the weight of the sample after immersion in PBS (gel component) and *W*0 is the initial weight of the sample.

#### 4.4.4. Moisture Content

Pre-cut samples were initially weighted and subjected to drying in an oven at 55 °C. To check moisture values for a determined period of time (24 h), the hydrogel membranes were weighed after 2 h, 4 h, 6 h, 8 h, and 24 h. Moisture content was determined at each analysis time using the following formula:

(4) $$MO\;\left(\%\right)=\frac{\left(Wi-Wd\right)}{Wi}\times 100$$

where *Wi* is the weight of the moist sample and *Wd* is the weight of the oven-dried sample.

#### 4.4.5. Moisture Uptake

Samples were cut at 1.5 cm diameter, weighed, and dried at 100 °C for 2 h. Then, they were weighed again and transferred into a desiccator for 24 h until reaching a constant weight. A supersaturated solution of NaCl was prepared and left inside the desiccator until 75% humidity was reached (verified with a thermohygrometer). The hydrogel membranes were incubated in the high humidity chamber for 72 h, after which they were weighed again, and the moisture uptake (*MU*%) was calculated according to the formula:

(5) $$MU\;\left(\%\right)=\frac{\left(Wm-Wi\right)}{Wi}\times 100$$

where *Wm* is the sample weight after 72 h in 75% humidity and *Wi* is the constant weight.

#### 4.4.6. Protein Adsorption

Firstly, a calibration curve using human serum albumin (HSA) was obtained, with the following concentrations: 0 µg/mL; 10 µg/mL; 20 µg/mL; 30 µg/mL; 40 µg/mL; 50 µg/mL; 60 µg/mL; 70 µg/mL; 80 µg/mL; 90 µg/mL; and 100 µg/mL. Then, three pieces of each membrane were cut at a default diameter of 1.5 cm and immersed in a 50 µg/mL solution of HSA in PBS and incubated in a shaking incubator at 37 °C for 24 h. After the incubation, 100 µL of each sample was mixed with 900 µL of Coomassie protein assay reagent. Absorbance was read at 595 nm, and the results were presented as mg of protein per gram of hydrogel. Firstly, we determined the amount of protein in the solution according to the mean value of the absorbance (triplicates) using the previously obtained calibration curve; then, we determined the amount of protein adsorbed by subtracting the value obtained for the sample from the HSA control. The values were converted to mg and expressed per gram of hydrogel.

#### 4.4.7. Inhibition of Protein Denaturation

Firstly, working solutions of 5% BSA in PBS and 500 µg/mL acetylsalicylic acid in purified water were prepared. Then, control samples and hydrogel membrane samples were obtained according to the quantities in Table 7. All samples were subjected to thermal denaturation at ~130 °C for 1 h.

After incubation, samples were vortexed for 1–2 min; then, large pieces of hydrogel were sedimented using a minicentrifuge for 30 s. Sample turbidity was read using the module 600 OD of the DeNovix Nanodrop spectrophotometer, supplied by DeNovix Inc., Wilmington, DE, USA, in 10 mm cuvettes. Samples with gel without protein were also prepared and used to subtract gel turbidity from the results and minimize errors. Samples were prepared in triplicate, and the results are expressed as mean (n = 3). The inhibition percent was calculated according to the formula:

(6) $$Inhibition\;\left(\%\right)=\frac{Ac}{\left(1-As\right)}\times 100$$

#### 4.4.8. Hydrolytic Degradation

The analysis was performed using a method previously described by Fuoco et al. [82], with slight modifications. Briefly, hydrogel membranes were cut at a diameter of 1.5 cm, and three pieces of each membrane type were dried in an oven at 60 °C until their weight was constant. Then, the samples were immersed in 10 mL of PBS (pH 7.4) and analyzed at different time periods: 0.5 h, 2 h, 4 h, 8 h, and 24 h. At each time, samples were dried for at least 3 h at 50 °C and then weighed. The final weights for each sample type were calculated as the mean from the three values, and the hydrolytic degradation percent was obtained using the following formula:

(7) $$Mass\;loss\;\left(\%\right)=\frac{\left(m0-mt\right)}{m0}\times 100$$

### 4.5. Hydrogel Membrane Morphological and Mechanical Characterization

#### 4.5.1. SEM Analysis

The surface morphology was evaluated by scanning electron microscopy (SEM) using Nova NanoSEM 630 with a UHR detector (Through-Lens-Detector-TLD), provided by FEI Company, Hillsboro, Oregon, USA, at an acceleration voltage of 10 kV. For porosity evaluation, the hydrogen membranes were swollen and incubated for 24 h in the freezer at approximately −20 °C prior to lyophilization.

#### 4.5.2. Rheological Studies

We subjected the gels to rheological characterization using a Kinexus Pro rheometer provided by Nexus Analytics (Malver, UK). The rheometer featured roughened geometry, with the lower plate measuring 50 mm and the upper plate measuring 40 mm. The distance between the geometrics was between 1.25 and 1.65 mm. The temperature was maintained using a Julabo CF41 cryo-compact circulator, provided by JULABO GmbH, Seelbach, Germany. In order to determine the linear viscoelastic region (LVER), amplitude sweep stress experiments were made at a constant frequency of 1 Hz and a temperature of 25 °C. For the frequency sweep stress measurements, the shear stress was maintained at 5 Pa (as determined from LVER), while the frequency was modulated between 0.1 and 50 Hz.

### 4.6. Antibacterial Effect Evaluation

Bacterial strains were streaked on Muller Hinton agar and incubated at 37 °C for 24 h prior to analysis. The analysis was carried out by disc diffusion technique, as follows. Firstly, suspensions of 0.5 McFarland were prepared, and two plates per microorganism have been inoculated. Then, the pre-cut samples (ø 0.5 cm) were carefully placed on each plate, and the doxycycline disc was added. Reading was performed after 24 h incubation at 37 °C.

### 4.7. In Vitro Cytotoxicity Tests

Murine fibroblast cells (NCTC, clone L929), human dermal fibroblasts (HDF), and human keratinocytes (HaCaT) were used to evaluate in vitro cytotoxicity of Ulvan-based hydrogels. Cells were grown in specific media (NCTC, clone L929, in Minimum Essential Medium (MEM), HDF in Dulbecco’s Modified Eagle Medium (DMEM), and HaCaT in RPMI 1640 medium) supplemented with 10% fetal bovine serum (FBS) and 1% antibiotics (penicillin, streptomycin, and neomycin) and maintained at 37 °C in a humidified atmosphere with 5% CO_2_. Hydrogels were cut into pieces of 5 × 5 mm^2^ and sterilized under UV light for 4 h. Subsequently, sample extracts were prepared by adding a piece of 5 × 5 mm^2^ in 500 µL of specific culture medium and incubated at 37 °C for 24 h. For in vitro tests, cells were seeded in 500 µL culture medium at a density of 5 × 10^4^ cells/mL in 24-well cell culture plates, and, after 24 h of incubation, the sample extracts were added. After cell incubation in standard conditions for 24 h and 48 h, the cell viability was assessed by the quantitative 3-(4,5-dimethylthiazol-2-yl)-2,5-diphenyltetrazolium bromide (MTT), assay and cell morphology was evaluated by fluorescence microscopy using the Live/Dead test [83].

For cell viability evaluation, treated cells were incubated with 0.25 mg/mL MTT solution for 3 h at 37 °C, followed by the dissolution of the insoluble formazan crystals with isopropanol. After 15 min of cell incubation at room temperature with gentle shaking for color uniformity, the absorbance was recorded at 570 nm using the microplate reader SPECTROstar^®^ Nano (BMG Labtech, Craiova, Romania). The amount of formazan was directly correlated to the number of metabolically active cells. The results were expressed as percentage of viability compared to the control (cells cultivated in culture medium alone), considered to have a viability of 100%. Data were presented as the average of three replicates (mean ± SD).

Cell morphology was assessed by fluorescence microscopy using LIVE/DEAD cell viability/cytotoxicity Kit (Molecular Probes, Invitrogen, Waltham, Massachusetts, USA) according to the manufacturer’s instructions. Briefly, after 48 h of cell incubation in standard conditions in the presence of hydrogel extracts, cells were washed with PBS and stained with calcein-AM (2 μM) and ethidium homodimer-1 (4 μM) at room temperature for 30 min. Fluorescent images were acquired using a Zeiss Axio Observer D1 microscope, provided by Zeiss, Jena, Germany, and further processed with ImageJ 1.51 software.

### 4.8. Scratch Wound Assay

Scratch assay was performed to evaluate the ability of Ulvan-based hydrogels to induce cell proliferation and migration into a wounded monolayer. The HaCaT keratinocytes were seeded in 24-well culture plates at a cell density of 3 × 10^5^ cells/mL and grown to confluence at 37 °C in a humid atmosphere with 5% CO_2_. Then, a linear wound was created in the cell monolayer with a sterile pipette tip, and the detached cells were removed by gentle washing with PBS. The sample extracts were added, and cells were further incubated in standard conditions for 24 h. Light microscope images were obtained at the beginning of the experiment (t = 0) and after 24 h of incubation using a Zeiss Axio Observer D1 microscope and AxioVision 4.6 software (Carl Zeiss, Jena, Germany) in order to evaluate the cell migration and covering of the injured area. Digital images were analyzed using ImageJ 1.51 software in order to quantify the cell migration rate (%). Samples were run in triplicate. The results were expressed as the mean value ± standard deviation of the recovery rates of the injured area. Statistical analyses were performed using Student’s t-test, with differences being considered statistically significant at *p* ≤ 0.05.

## Figures and Tables

**Table 1:** Properties of different materials used for wound healing applications.

Material		Composition	Advantages	Disadvantages	Ref.
Natural polymers	Chitin	Semicrystalline polysaccharide made up of monomeric units of—(1→4)-2-amino-2-deoxy-d-glucose and—(1→4)-2-acetamide-2-deoxy-d-glucose.	Gel-forming Biodegradable Non-toxic Antimicrobial properties	Weak mechanical properties Poor solubility in aqueous solutions	[22,23]
	Chitosan	Co-polymers *N*-acetyl-d-glucose amine and d-glucose amine	Gel-forming Biodegradable Non-toxic Antimicrobial properties Surface-induced thrombosis, platelet activation, and blood clotting Cytokine release	Solubilization in acetic acid solution, toxic for mammalian cells Hydrogels made from chitosan and crosslinking agents have less antimicrobial effect due to poor solubility Properties vary depending on origin, extraction method, and molecular weight.	[24]
	Collagen	Amino acids proline, glycine and hydroxyproline	Anti-inflammatory Antifibrotic Analgesic properties Promotes angiogenesis	Denaturation in the manufacturing process can determine loss of therapeutic effect Lack of antimicrobial effect Weak mechanical properties	[25]
	Carrageenan	Sulphated polysaccharide composed of alternative units of 3-linked b-d-galactopyranose (G-units) and 4-linked a-d-galactopyranose (d-units)	Good viscoelastic properties Water soluble Low cytotoxicity Antimicrobial and antioxidant properties Does not stick to the wound	Low mechanical strength Difficult reproducibility because the gel network is influenced by many factors (ions, molecular weight, monosaccharide content, pH, and temperature Can have side effects on blood, clothing, and immune system	[26,27]
	Silk fibroin	Fibrous protein with a semi-crystalline structure, with a heavy and a light chain, linked through a disulfide bound	Highly biocompatible and biodegradable Increase expression of NF-kB signaling pathway, which influences cell proliferation and adhesion, as well as inflammation Determines expression of fibronectin, type III collagen, MMP-12, and integrin β1 in burn-induced rat wounds	No antimicrobial effect Bacterial biofilms can form on the surface of silk fibers	[28]
	Cellulose	Organic polysaccharide, composed of d-glucose bound by 1-4-β-glycosidic linkages	Can be obtained from various sources (bacteria, tunicates, and plants) Has tunable physico-chemical, mechanical, and biological properties Is biocompatible and biodegradable	Cannot be used in its native form because it is rich in hydroxyl groups Lack of antibacterial effect	[29]
	Alginate	Composed of β-d-mannuronate (M) and α-l-guluronate residues, linked by 1,4 glycosidic bonds	Adsorbent properties Can form a gel with wound exudate Can keep a moist environment Hemostatic effect Can be easily remove, without inducing further trauma	Their lack of adhesive properties triggers the need for a second dressing Can trigger allergic reactions	[30]
	Hyaluronic acid	High molecular weight polysaccharide, composed of d-glucuronic acid and *N*-acetyl glucosamine	High biocompatibility No immunogenicity Stimulates cell migration, proliferation, and differentiation Regulates the expression of extracellular matrix proteins Contributes to the removal of dead tissue Induces secretion of inflammatory cytokines Regulates the re-epithelization process by interacting with keratinocyte CD44 receptors	Low mechanical stability Easily adheres to skin and can cause additional damage when removed Needs to be modified to be suitable for wound dressing	[31]
	Ulvan	Sulfated polysaccharide, consisting of glucuronic acid and/or iduronic acid coupled with sugars such as rhamnose, xylose, arabinose and glucose	Water soluble Antioxidant and antimicrobial activity Immunomodulatory action Improved swelling rate Rhamnose-rich polysaccharides can trigger cell proliferation and collagen synthesis Only algal polysaccharide that contains iduronic acid, which has similar structure to mammalian glycosaminoglycans (GAG) Utilizing algal compounds can contribute to the global problem of nuisance macroalgal blooms	High variability in composition due to algal habitat, season of collection, and extraction methods, which can induce modified bioactivity. Poor mechanical strength.	[32,33]
Synthetic polymers	Polyvinyl alcohol (PVA)	PVA is obtained through the hydrolysis or alcoholysis of polyvinyl acetate (PVAc). The process typically involves base-catalyzed hydrolysis in the presence of methanol or ethanol.	Non-carcinogenic nature, bio-adhesive properties, non-toxicity, and exceptional film-forming ability and transparency, as well as a high swelling capacity in water or biological fluids	PVA hydrogel has inadequate elasticity	[34,35]
	Polyurethane (PU)	PU is obtained by the condensation polymerisation of an isocyanate (R′-(N=C=O)n) and a polyol (R-(OH)n). The most commonly used isocyanates are the aromatic diisocyanates toluene diisocyanate (TDI) and methylene diphenyl diisocyanate (MDI) and the most commonly used polyols are hydroxyl-terminated polyethers or polyesters.	High biocompatibility, high mechanical strength and flexibility, easy processability, possibility to modify the surface functional properties	PU has hydrophobic properties; the hydrophilicity must be improved. PU has no antibacterial properties and may be toxic due to the obtaining process by using petroleum-based raw material.	[36,37]
	Polyvinylpyrrolidone (PVP)	PVP is a nonionic polymer compound produced by the polymerization of N-vinylpyrrolidone (NVP).	Biodegradable, water soluble, biocompatible, antibacterial mechanism. PVP has the capacity to encapsulate and release antibiotics.	Hydrogel blends with higher PVP concentrations exhibit increased fragility and rapid dissolution in water, which can limit their stability and effectiveness in long-term applications. PVP has inferior mechanical properties and limited swelling capability.	[38,39,40]
	Polycaprolactone (PCL)	PCL is a polyester that can be obtained by two methods: (a) ring opening polymerization of ε-caprolactone by using stannous chloride or (b) polycondensation of 6-hydroxyhexanoic acid (C_6_H_12_O_3_).	Biocompatibility, biodegradability, bioresorbable, FDA approved, and has slower degradation rate. Electrospun PCL fibers are well-suited for the treatment of both acute and chronic wounds.	PCL has limited antimicrobial activity, and its highly hydrophobic nature provides fewer binding sites for cell adhesion, migration, and proliferation.	[41,42]
	Poly(lactide-co-glycolide) (PLGA)	PLGA is obtained by ring-opening co-polymerization of lactic acid (LA) and glycolic acid (GA). During polymerization, monomeric units of LA and GA are sequentially connected in PLGA through ester linkages, resulting in the formation of a linear, aliphatic polyester.	Good mechanical strength, biocompatible, biodegradable, FDA approved. PLGA is used in the preparation of skin substitutes. PLGA hydrogels are used for controlled delivery of various therapeutic agents.	PLGA-based scaffolds are hydrophobic and semi-permeable, which restricts their ability to absorb exudates. The degradation of PLGA into its monomers can lead to their accumulation in the body, potentially causing localized pH fluctuations and inflammation. Excessive acidity from PLGA degradation can trigger apoptosis or necrosis, damaging surrounding tissues.	[41,42,43]

**Table 2:** Percentage (g/%) of carbon, hydrogen, nitrogen, and sulfur for Ulvan.

Chemical Element	Percentage (g/%)
Carbon	25.9
Hydrogen	5.0
Nitrogen	3.3
Sulfur	4.2

The measurements were performed in duplicates.

**Table 3:** Proportion (%) of monomers in methanolic hydrolysate of Ulvan.

Monomers	Proportion (%)
Rhamnose	48.7
Glucuronolactone	24.8
Glucose	11.3
Xylose	4
Ribose	3.5
Galactose	2.38
Mannose	1

**Table 4:** The variation of pH among different hydrogel batches.

Sample	pH Value (*n* = 3)	Average	STDEV	CV
Base (Alg + PVA)	6.59	6.66	0.06	0.92
	6.71			
	6.67			
0.1 ULV	6.89	6.73	0.23	3.38
	6.83			
	6.47			
0.5 ULV	6.89	6.94	0.07	0.98
	6.92			
	7.02			
1 ULV	7.15	6.72	0.63	9.37
	7.02			
	6.00			

The measurements were performed in triplicate.

**Table 5:** Rheological parameters of hydrogels.

Sample	G′ (Pa)	G″ (Pa)	Yield Stress (Pa)
Alg + PVA	7071	621.8	1584
Alg + PVA + 0.1 ULV	8818	773.2	1294
Alg + PVA + 0.5 ULV	9322	717.1	631.2
Alg + PVA + 1.0 ULV	16,050	1583	2049

**Table 6:** Average pore size data for all hydrogels evaluated.

Sample	Min–Max (µm)	FWHM (µm) Full Width at Half Maximum	Mean ± SD (µm)
Alg PVA	3–120	12–51	30.8 ± 21.0
0.1 ULV	7–112	15–50	32.5 ± 17.8
0.5 ULV	16–131	29–66	48.1 ± 19.3
1 ULV	20–186	35–90	64.2 ± 29.1

**Table 7:** Preparation of samples for protein denaturation inhibition study.

Sample/Control	BSA Solution Quantity	Gel/Water Quantity	PBS Quantity
Positive control (aspirin)	900 µL	200 µL aspirin solution	2800 µL
Negative control	900 µL	200 µL water	2800 µL
Hydrogel membrane samples	900 µL	200 mg	2800 µL

## Data Availability

The original contributions presented in this study are included in the article. Further inquiries can be directed to the corresponding authors.
